# Low-cost bodyweight callisthenic-based HIIT improves blood pressure, glucose control, and TyG in older adults with metabolic syndrome

**DOI:** 10.3389/fspor.2026.1655441

**Published:** 2026-02-11

**Authors:** Fabio Nascimento-da-Silva, Angela Maria Moed Lopes, Renata Alves Andrade Moreira Araujo, João Pedro Werneck-De-Castro, João Rafael Valentim-Silva

**Affiliations:** 1 Pitágoras UNOPAR Anhanguera University, Londrina, Brazil; 2 MUST University, Boca Raton, FL, United States; 3 Division of Endocrinology, Diabetes and Metabolism, University of Miami, Miami, FL, United States; 4 Federal University of State of Rio de Janeiro, Rio de Janeiro, Brazil; 5 Federal University of Santa Catarina, Florianópolis, Santa Catarina, Brazil; 6 University of Vassouras, Saquarema, Brazil; 7 Education and Technology College of Amazon, Abaetetuba, Pará, Brazil; 8 Medicine Department, Araruama College, Araruama, Rio de Janeiro, Brazil

**Keywords:** aging, exercise training, metabolic health, metabolic syndrome, metabolism

## Abstract

**Objective:**

Lack of time is the main barrier to physical exercise routines. This study evaluated the impact of whole-body, low-cost, high-intensity interval training (HIIT) using bodyweight exercises on old subjects with metabolic syndrome (MetS).

**Material and methods:**

Forty participants (mean age: 72.4 ± 5.9 years; 36 women) with MetS admitted to a public health service center were randomized into control and HIIT groups. The 40-min HIIT protocol consisted of 60-s exercises at 75%–85% maximum heart rate monitored by finger oximeter, followed by 120 s of passive rest, performed three times weekly for eight weeks. We assessed exercise capacity (VO_2_max), blood pressure, biochemical parameters, and the triglyceride-glucose (TyG) index at baseline, four and eight weeks after exercise. The TyG index was used as a biomarker for insulin resistance and cardiometabolic health, and the MetS-Z to Metabolic Syndrome to severity of this condition.

**Results:**

HIIT improved VO_2_max (∼20%), reduced systolic and diastolic blood pressure, fasting glucose (−32%), HbA1c (−35%), triglycerides (−39%), and TyG index (−12%) compared to controls.

**Conclusions:**

The low-cost bodyweight HIIT protocol is an easy and effective strategy for improving cardiometabolic health in the elderly with MetS. Our results highlight the importance of introducing this approach into public health programs, particularly in resource-limited settings, to manage metabolic disorders and promote healthy aging.

## Introduction

Metabolic syndrome (MetS) is a complex and multifactorial condition characterized by three or more risk factors, such as elevated blood pressure, impaired glucose metabolism, non-alcoholic fatty liver disease, diabetes, excessive visceral fat, and dyslipidemia (1, 2). MetS is associated with an increased risk of comorbidities and mortality (3), and the prevalence of MetS among adults ranges from 15% to 35%. Although the cause of MetS is still debatable, insulin resistance (IR) and visceral obesity are postulated as the main triggering causes (1). Genetic predisposition, sedentary lifestyle, smoking, and poor diet also contribute to the development and progression of the disease (4–6). In addition, aging increases the risk of developing MetS. Therefore, it is imperative to find efficient interventions to prevent and treat individuals suffering from MetS.

Lack of physical exercise (PE) is a major cause of chronic diseases (7). PE provides a physiological stimulus that evokes a coordinated response and crosstalk between multiple organs and systems. PE offers wide-ranging health benefits when repeated regularly (i.e., training) and reduces the risk of all-cause mortality (8–10). PE training helps prevent MetS and its associated comorbidities. Multiple types of structured PE enhance health in people with hypertension, cardiovascular disease, obesity, and diabetes (11–14). Traditionally, PE interventions have focused on moderate-intensity continuous training (MICT) and resistance training (14). Current recommendations state that the general population and adults with metabolic diseases should engage in at least 150–300 min of moderate to vigorous-intensity aerobic activity weekly and resistance training 2–3 days a week (14–16). Alternatively, guidelines suggest that shorter durations (75 min/week) of vigorous-intensity or interval training are sufficient to promote the beneficial metabolic effects (14–16).

High-intensity interval training (HIIT) training is a regimen that involves exercise training done at high intensities (65%–90% V˙O2 peak or 75%–95% heart rate peak) for a short period (10 s to 4 min) with 12 s to 5 min of active or passive recovery (17). Recently, HIIT has been proposed as an exercise modality with similar or better benefits to the cardiovascular system and whole-body metabolism than MICT (11, 12, 18–21). Randomized controlled trials and meta-analyses have demonstrated HIIT's efficacy not only in healthy sedentary individuals but also in patients with cardiometabolic disorders, including type 2 diabetes, obesity, hypertension, and metabolic syndrome. For example, HIIT improves insulin sensitivity, blood pressure, and lipid profiles in adults with MetS (22, 23) as well as enhances VO_2_max and quality of life in individuals with chronic conditions such as heart failure and type 2 diabetes (11, 12). These findings establish HIIT as a robust and adaptable training modality across multiple at-risk populations. A noteworthy advantage of HIIT over MICT is that HIIT involves intense short exercise sessions, reducing the time required for exercise sessions. Indeed, HIIT is more efficient than MICT in managing glucose metabolism and lipid profiles in old subjects (21, 24, 25). Additionally, HIIT improves several biochemical, hematological, and physiological markers related to metabolic diseases (17, 26, 27). Clinically important, although HIIT is performed at high exercise intensity, it is safe for several populations, including the elderly (28, 29).

HIIT can be applied to any exercise, such as running, resistance, and swimming. Most HIIT interventions rely on equipment such as treadmills, cycle ergometers, or resistance machines, which may not be accessible to older adults in resource-limited settings. Exercises using solely body weight as an external load to induce health benefits are practical, low-cost, and versatile compared to exercises performed with equipment and devices (18). This allows exercise practice in various settings, including homes and parks, which is especially important for low-income and elderly populations with limited access to exercise facilities and equipment. Therefore, developing low-cost and effective exercise routines is necessary. However, the literature regarding the effects of bodyweight HIIT on MetS is scarce, particularly in old individuals.

Because the cardiometabolic benefits of HIIT in metabolic disorders are well established, the primary purpose of the present study was not to demonstrate HIIT superiority over other exercise modalities, but to test the pragmatic feasibility and real-world effectiveness of a standardized, low-cost, bodyweight-only HIIT protocol delivered within primary care. In addition, we incorporated the TyG index and MetS-Z to provide clinically relevant, integrated markers of insulin resistance and metabolic syndrome severity that may be particularly informative for public health implementation.

## Materials and methods

### Participants

The Institutional Review Board (IRB) of the Brazilian National Health Council approved the study (# CAAE 02279512.1.0000.0012), under number 025794/2012, and based on the National Law 466/2012, and following the ethical principles of the Declaration of Helsinki. All participants signed the consent form after receiving verbal instruction and before any intervention. The volunteers were seen by a physician who released them to perform vigorous exercise before participating in the study. The volunteers were recruited to the study after checking into one of the public Basic Health Centers of the City of Porto Velho, Rondônia, Brazil.

The intervention was conducted at a public Basic Health Center. The environment was not climate-controlled; however, since the assessments took place in the morning, the conditions were adequate. Measurements obtained with a thermometer and thermo-hygrometer indicated average values of 26–28 °C and approximately 70% relative humidity every day. The region where the study was carried out is characterized by about six months of dry season and six months of rainfall, which contributes to variations in air quality. All procedures followed the guidelines of the Brazilian Unified Health System (SUS), emphasizing accessibility and low-cost interventions.

**Figure 1:**
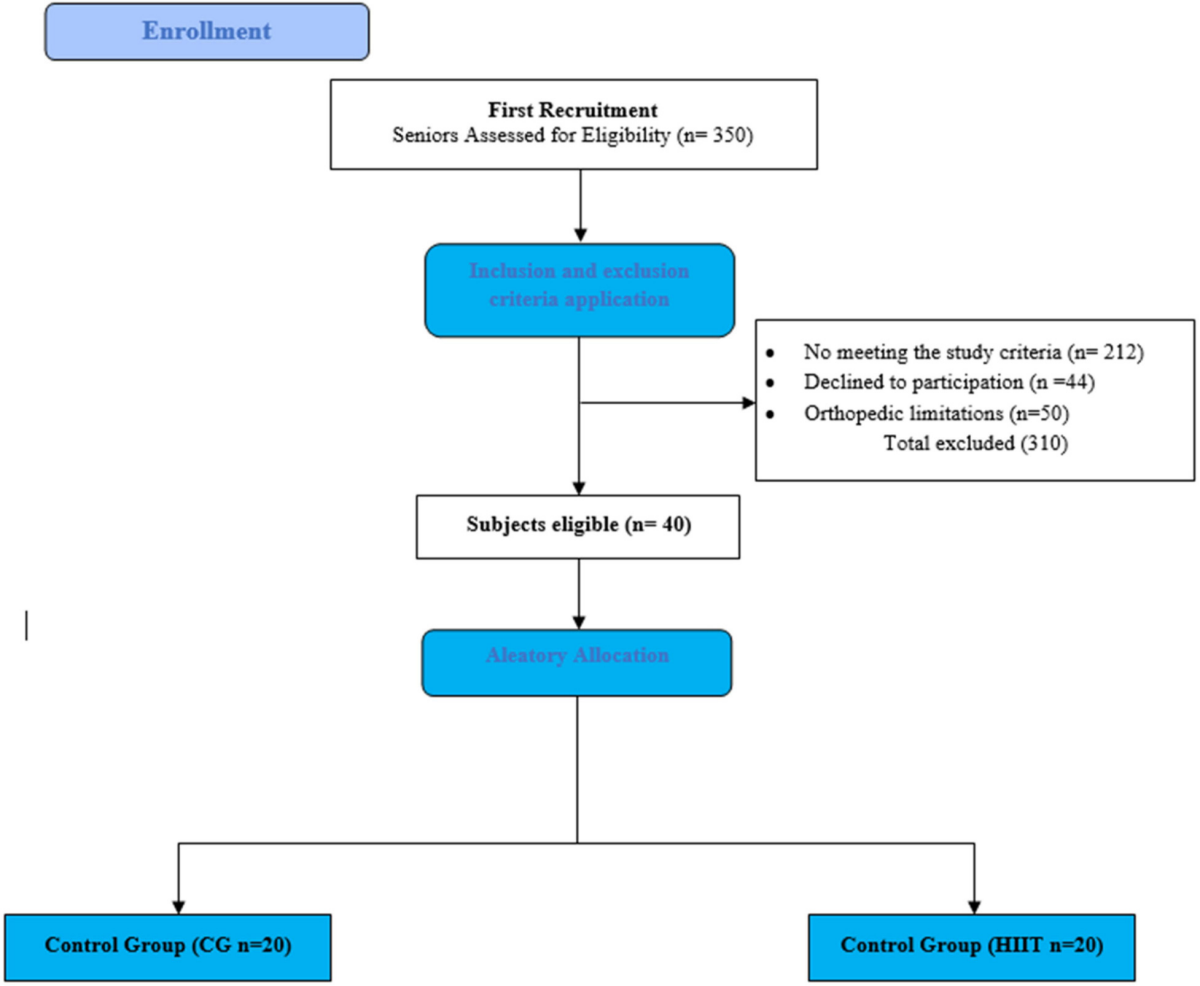
Consort flow chart. Selection and allocation process of volunteers.

No volunteer participated in sports or physical training regularly. Metabolic syndrome (MetS) was determined based on the criteria defined by the World Health Organization (WHO) and the National Heart, Lung, and Blood Institute of the United States National Institutes of Health (NIH) (30). Only subjects exhibiting three out of the four criteria described in Table 1 and being 60 years or older were included in the study. Subjects presenting any orthopedic limitation to the exercise training were excluded from the study. Figure 1 shows the consort flow chart for subject's selection.

**Table 1:** Criteria for clinical diagnosis of metabolic syndrome.

Measure	Cutoff
Waist circumference^a^	>102 cm to males >88 cm to females
Triglycerides	>150 mg/dL (1.7 mmol/L)
HDL-C	<40 mg/dL (1.0 mmol/L in males) <50 mg/dL (1.3 mmol/L in females)
Blood pressure	Systolic >130 and/or diastolic >85 mm/Hg
Fasting glucose	>100 mg/dL

HDL-C, high-density lipoprotein cholesterol.

a Reference for Brazilian population (31).

### Study design

Forty subjects, 36 females and four males, were enrolled and randomly divided into the control group (no exercise; *n* = 20) or HIIT (*n* = 20). Before the study, participants had the opportunity to familiarize themselves with all the procedures on two occasions. First, they visited the laboratory after fasting for 12 h and refraining from caffeine, alcohol, and exercise for 24 h. Body mass, height, and blood pressure were assessed. Baseline blood samples were obtained to determine hematological and biochemical parameters. In the second visit (4 days later), subjects of the HIIT group were familiarized with the HIIT protocol. After the familiarization sessions, baseline measurements of maximal exercise capacity were assessed.

Participants were allocated 1:1 to HIIT or control using computer-generated random numbers and, after, the allocation was conducted in accordance with the result. Due to the nature of the intervention, participants and trainers were not blinded; however, outcome assessors and laboratory staff were blinded to group allocation whenever feasible.

All evaluations were conducted at a public Basic Health Center, between 7:00 and 9:00 a.m. They were instructed to fast for 8–10 h, avoid alcohol for 24 h, and refrain from vigorous physical activity for 24 h prior to each assessment. Subjects were followed up four and eight weeks after initiating the HIIT protocol.

### Body composition and blood pressure assessment

Body weight*,* waist circumference, and body mass index (BMI), were assessed using the octopolar scale (InBody, model 170, South Corea) (32). Blood pressure was determined following the previously described American Heart Association (AHA) procedures (33). The measurement was always performed on the right arm after 5 min of resting on a chair and using a sphygmomanometer and stethoscope (PA.MED, Brazil). The blood pressure was assessed by a very experienced nurse from the Basic Health Center.

### Blood sampling, biochemical and hematological assessment

Blood samples were obtained by venipuncture and collected in EDTA-containing tubes for plasma and tubes with gel to separate the serum (BD Vacutainer, Franklin Lakes, NJ, USA). Hematocrit, hemoglobin, mean corpuscular volume, and mean corpuscular hemoglobin were analyzed using the Hemocauto Analyzer 2120® (Siemens, AG, Erlangen, Germany). Blood glucose, glycosylated hemoglobin, and high-density lipoprotein C were assessed with the automatic device Konelab 60i after calibration. The coefficient of variation was 1.2% to red globule count, 0.93% to hemoglobin, and 2.93% to plaquette count. All analysis was conducted in duplicates. All measurements were performed until 60 min after the blood collection.

### Exercise capacity

To determine maximal exercise capacity, volunteers underwent the 6-minute walking test (6MWT). The test was performed outdoors, and participants were instructed to walk at their own pace and alone, covering as much distance as possible within 6 min. They were allowed to stop and rest, if necessary, before resuming (34).

All individuals were advised to wear light clothing and to refrain from exercise prior to the test**.** The following sex-specific equations were used to estimate cardiorespiratory fitness from the 6-min walk test (6MWT), expressed as estimated VO_2_max (mL·kg^−1^·min^−1^):

$$\begin{array}{rl} & \mathrm{Men}:\mathrm{estimated}\;VO_{2\max}=(7.57\times \mathrm{height}[\mathrm{cm}])\\ & \;-(5.02\times \mathrm{age}[\mathrm{years}])-(1.76\times \mathrm{body}\;\mathrm{mass}[\mathrm{kg}])-309\\ & \mathrm{Women}:\mathrm{estimated}\;VO_{2\max}=(2.11\times \mathrm{height}[\mathrm{cm}])\\ & \;-(2.29\times \mathrm{body}\;\mathrm{mass}[\mathrm{kg}])-(5.78\times \mathrm{age}[\mathrm{years}])+667.6 \end{array}$$

### High-intensity interval training protocol

The HIIT program involved bodyweight exercises, requiring no specialized equipment besides exercise mats and small step boxes. Exercise sessions lasted 40 min including 5 min warmup and cool-down. Participants were instructed on proper form and safety during a pre-study familiarization session led by certified trainers. Warm-up included dynamic stretching (arm swings, leg swings, trunk rotations) and light calisthenics (marching in place, low intensity jumping jacks). The protocol was standardized across sessions, ensuring consistency in intensity and execution. Each of the 10 exercises was performed once for 60 s with 120 s of passive recovery between exercises. Passive recovery consisted of sitting quietly or standing still.

Although each exercise was performed for 60 s, totaling 20 stimuli per session (five exercises repeated four times), not all exercises were performed in every daily session. Thus, the effective duration of high-intensity effort was estimated to be approximately 15–18 min, representing the sum of the active period and the time during which heart rate remained elevated throughout the recovery phase.

The cooldown included 5 min of breathing and stretching exercises. Participants performed the HIIT sessions three times a week for eight weeks. Adherence to the protocol was monitored weekly using attendance logs, and participants were instructed to maintain their usual diet and lifestyle during the intervention. Additionally, they were advised to refrain from other structured physical activities to isolate the effects of the HIIT intervention.

Subjects performed a standardized warm-up of stretching and calisthenics exercises for 5 min before HIIT sessions. All HIIT sessions were conducted in the following exercise order: a) ten meters back and forth running, b) air squats, c) push-ups, d) sit-ups, e) jumping jacks, f) forward lunges, g) abdominal planks, h) reverse lunges, i) abdominal plank, j) side steps, direction-changing footwork and going up and down steps (25 cm). A 5-min cooldown was carried out at the end of the session with easy stretching and breathing exercises.

Exercise intensity during the HIIT sessions was monitored using heart rate measurements obtained every 5 s with a ROSSMAX finger oximeter. Maximal heart rate (HRmax) was estimated for each participant using the age-predicted formula (HRmax = 220 − age). Target heart rate zones for the HIIT protocol were defined as percentages of HRmax, with high-intensity intervals executed at >90% of HRmax to ensure vigorous effort consistent with published HIIT guidelines. This proceeding ensured that participants exercised within predetermined high-intensity zones, providing objective control of cardiovascular stress throughout the intervention.

### Insulin resistance assessment (TyG index)

The triglyceride–glucose (TyG) index was calculated using the following validated formula:

TyG=ln[(fastingtriglycerides(mg/dL)×fastingglucose(mg/dL))/2]

This formula yields a dimensionless score, with higher values indicating greater insulin resistance. A cutoff value of TyG ≥4.5 has been proposed to identify insulin resistance in adults. All values reported in this study were expressed consistently using this definition (35, 36).

### METS-Z score

To determine the effects of HIT on MetS parameters, we assessed the metabolic syndrome severity score (MetS-Z) (37). The MetS-Z is based on data from the National Health and Nutrition Examination Survey (NHANES) collected between 1999 and 2010, which represents a broad cross-section of the U.S. population. MetS-Z functions as a standardized z-score, normally distributed with a mean of 0 and a standard deviation of 1. In the context of this study, it indicates how many standard deviations a participant's MetS-Z deviates from the NHANES population average.

### Statistical analysis

**Figure 2:**
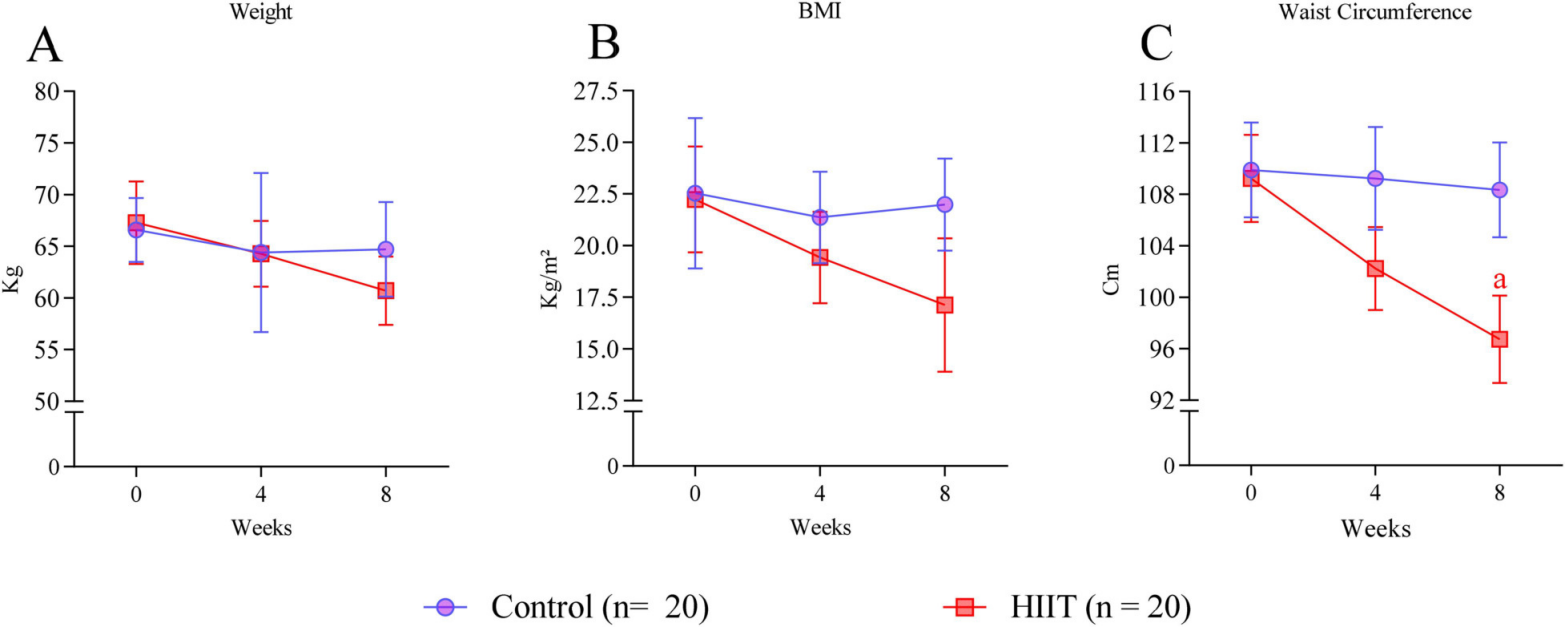
Body composition*.* **(A)** Body mass, **(B)** body mass index, **(C)** Waist-Circumference before and after 4 and 8 weeks of HIIT. Letter in the graph denote *p* < 0.05: *a* compared to baseline (week 0) determined by Two-way ANOVA followed by Sìdak *post-hoc* test.

**Figure 3:**
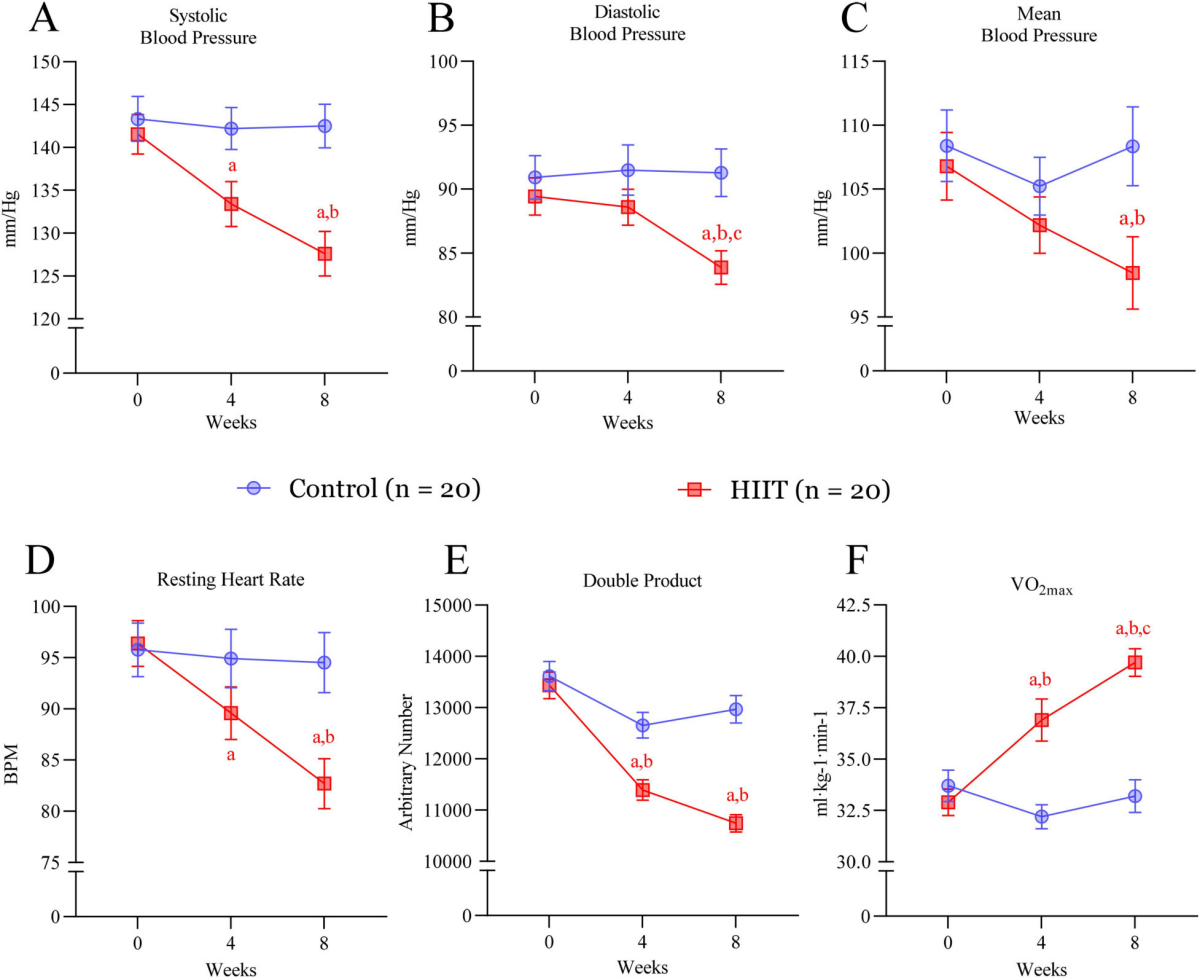
HIIT ameliorates cardiovascular parameters and increases exercise capacity. **(A)** systolic blood pressure, **(B)** diastolic blood pressure, **(C)** mean arterial pressure, **(D)** resting heart rate, **(E)** double product (systolic blood pressure × heart rate) and **(F)** exercise maximal oxygen consumption (VO2_max_) before (week 0) and after 4 and 8 weeks of intervention. Letters in the graph denote *p* < 0.05: *a* compared to baseline (week 0); *b* to control group and *c* to four weeks within the group and were determined by Two-way ANOVA followed by Sìdak *post-hoc* test.

Data are presented as mean ± standard deviation. Data normality was assessed using the Shapiro–Wilk test. Two-tail Student's *T*-test was used to compare means of control and HIIT groups before exercise (Baseline measurements; Table 2). Two-way ANOVA followed by Sìdak *post-hoc* test was used to determine differences between means within (over time) and between groups (Figures 1–3). Because an *a priori* sample size calculation was not performed, we report effect sizes and 95% confidence intervals for all primary outcomes. Statistical significance was set as *p* < 0.05 and assessed using GraphPad Prism 9.

**Table 2:** Age, anthropometric, physiological, and biochemical parameters of participants before intervention.

Measure	Control (*n* = 20)	HIIT (*n* = 20)	*p*
Age (years)	71.6 ± 6.7	73.1 ± 5.0	0.195
Body Weight (Kilograms)	66.6 ± 3.1	67.3 ± 4	0.117
Stature (meters)	1.72 ± 0.1	1.74 ± 0.2	0.151
Body Mass Index (Kg/m^2^)	22.5 ± 1.7	22.2 ± 1.4	0.752
VO_2_ Max (mL·kg^−1^·min^−1^)	32.2 ± 2.6	33.7 ± 3.4	0.272
VO_2_ Max (mL·min)	1.60 ± 0.4	1.63 ± 0.6	0.135
Triglycerides (mg·dL)	184.5 ± 38.7	186.1 ± 33.2	0.266
Glucose (mg·dL)	155.5 ± 11.6	157.3 ± 16.2	0.482
Glycosylated Hemoglobin (%)	9.2 ± 2.2	9.3 ± 2.2	0.674
Insulin Resistance (TyG Index)	6.7 ± 0.5	6.6 ± 0.7	0.129
Systolic Blood Pressure (mmHg)	141.5 ± 10.3	143.3 ± 11.7	0.542
Diastolic Blood Pressure (mmHg)	89.4 ± 6.5	90.9 ± 7.6	0.564
Waist-circumference			
Male	109.9 ± 6.0	109.2 ± 6.2	0.212
Female	89.3 ± 4.5	88.9 ± 6.2	0.081
HDL-C	39.5 ± 4.4	39.3 ± 5.3	0.908

HDL-C, high-density lipoprotein-C. Results shown as mean ± standard deviation. *P* value was determined by unpaired two-tail Student's *T*-test.

## Results

### Demographics and body composition

Anthropometric, physiological, and biochemical parameters were comparable between groups at baseline (week 0) (body mass: control vs. HIIT mean difference = 1.13 kg, 95% CI: −6.31 to 8.58; *p* = 0.7636; BMI: control vs. HIIT mean difference = 2.37 kg·m^−2^, 95% CI: −2.06 to 6.80; *p* = 0.2917; waist circumference: control vs. HIIT mean difference = 6.43 cm, 95% CI: 0.64–12.23; *p* = 0.0299). Detailed baseline data are presented in Table 2.

### Body composition

Eight weeks of HIIT did not decrease body weight or body mass index [no group × time interaction for body mass: *F*(2,114) = 0.15, *p* = 0.8616; and BMI: *F*(2,114) = 0.35, *p* = 0.7028] (Figures 2A,B). However, waist circumference was reduced at the end of the HIIT program [significant main group effect: *F*(1,114) = 4.84, *p* = 0.0299; mean difference = −6.43 cm, 95% CI: −12.23 to −0.64] (Figure 2C).

### Effects of HIIT on blood pressure and exercise capacity

The HIIT intervention resulted in significant improvements in cardiovascular parameters and exercise capacity. Systolic blood pressure, resting heart rate, double-product, and VO2max were improved significantly in the HIIT group compared to sedentary subjects at four weeks of training (SBP mean difference = −8.81 mmHg, 95% CI: −19.48 to 1.86; *p* = 0.203; VO_2_max mean difference = 4.70 mL·kg^−1^·min^−1^, 95% CI: 1.48–7.92; *p* = 0.0004) (Figure 3). After 8 weeks, the HIIT group showed reductions in systolic blood pressure by 9.4% (−14.90 mmHg, 95% CI: −25.57 to −4.23; *p* = 0.0009), diastolic blood pressure by 6.2% (−7.41 mmHg, 95% CI: −14.33 to −0.49; *p* = 0.0260), and mean arterial pressure had and tendency to decrease (−9.91 mmHg, 95% CI: −21.16 to 1.34; *p* = 0.134) (Figures 3A–C). While HIIT decreased resting heart rate (−11.80 bpm, 95% CI: −22.85 to −0.75; *p* = 0.0269) and the double product (−2,224 units, 95% CI: −3,251 to −1,197; *p* < 0.0001), it increased VO2max by 20% at the end of the intervention (+6.50 mL·kg^−1^·min^−1^, 95% CI: 3.28–9.72; *p* < 0.0001) (Figures 3D–F). In contrast, no changes were observed in the control group (Figure 3).

### Hematological assessments

**Figure 4:**
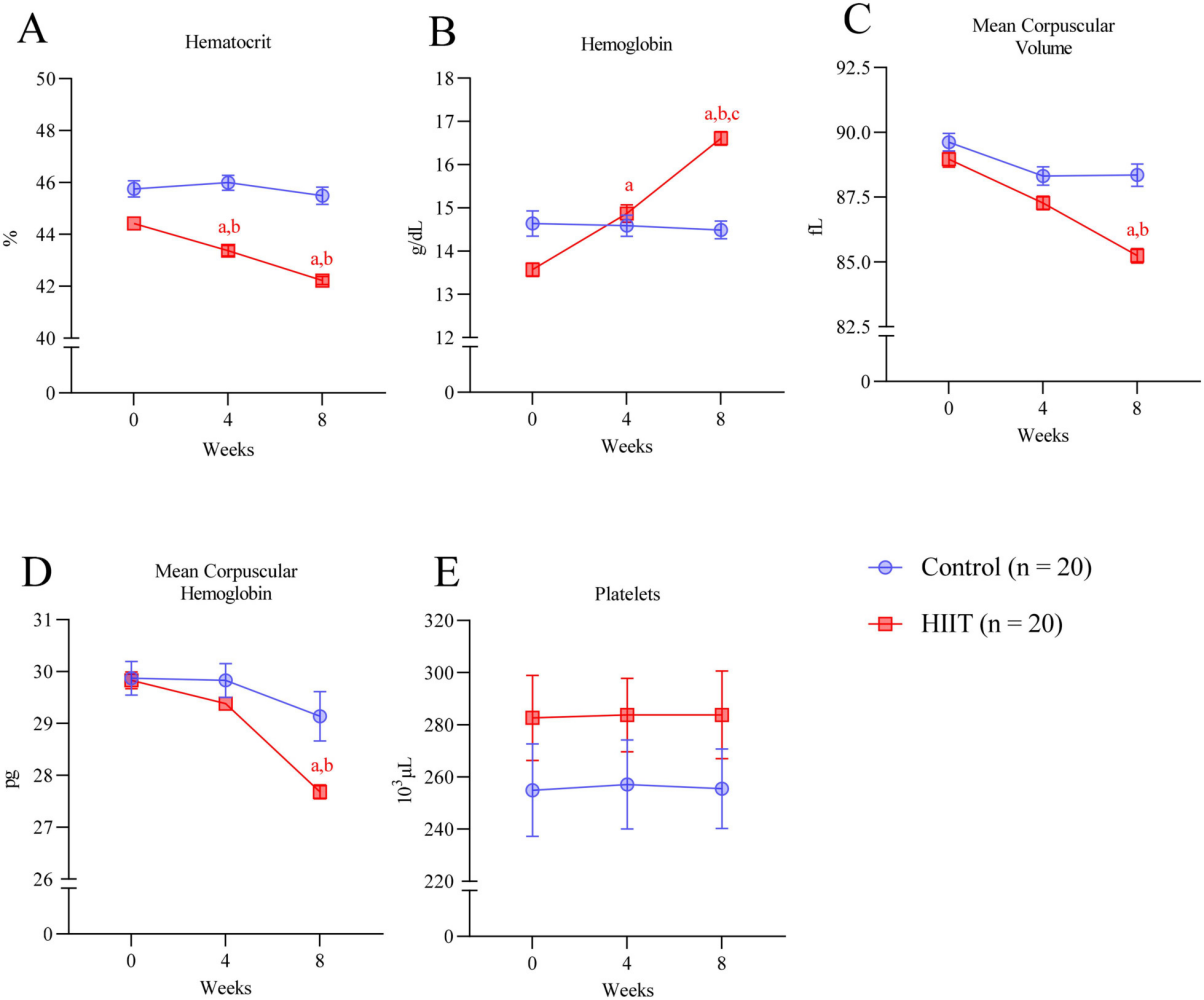
Hematological changes in response to HIIT. **(A)** Hematocrit **(B)** hemoglobin, **(C)** mean corpuscular volume, **(D)** mean corpuscular hemoglobin, and **(E)** platelet before (week 0) and after 4 and 8 weeks of intervention. Letters in the graph denote *p* < 0.05: *a* compared to baseline (week 0); *b* to control group and *c* to four weeks within the group and were determined by Two-way ANOVA followed by Sìdak *post-hoc* test.

HIIT induced moderate alterations in hematological markers. Hematocrit decreased by 4.9% (mean difference = −2.20%, 95% CI: −3.28 to −1.12; *p* < 0.0001) while hemoglobin increased by 7.7% (mean difference = +3.04 g·dL^−1^, 95% CI: 2.14–3.95; *p* < 0.0001) in the HIIT group after 8 weeks (Figures 4A,B). Mean corpuscular volume (mean difference = −3.72 fL, 95% CI: −5.13 to −2.32; *p* < 0.0001) and mean corpuscular hemoglobin (mean difference = −2.15 pg, 95% CI: −3.36 to −0.94; *p* < 0.0001) also declined significantly (Figures 4C,D). Platelet counts remained stable across both groups (HIIT vs. control at 8 weeks: mean difference = −28.32 × 10^3^ µL^−1^, 95% CI: −97.10 to 40.46; *p* = 0.976) (Figure 4E).

### Plasma glucose and triglycerides after HIIT

**Figure 5:**
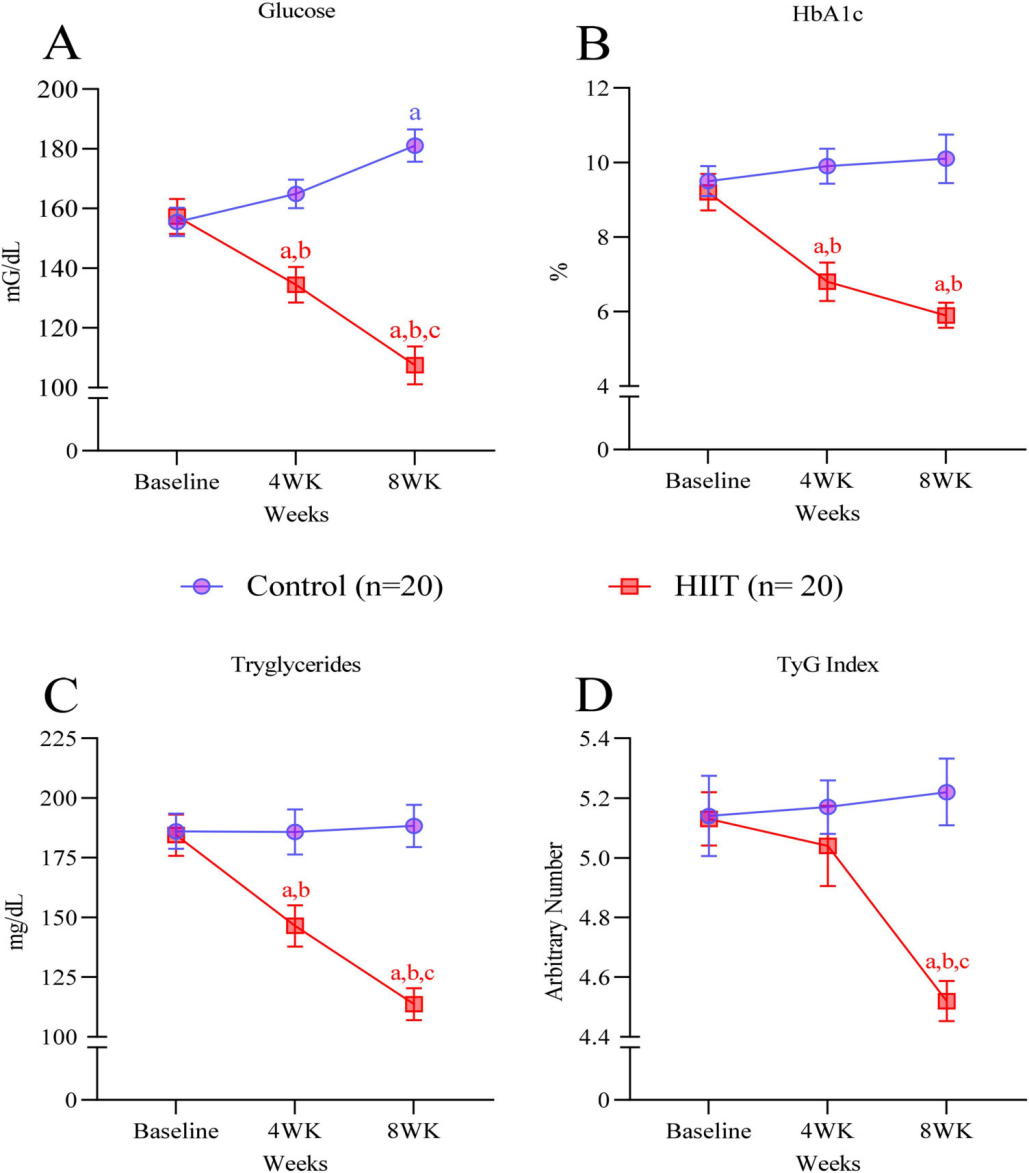
HIIT decreases plasma glucose and triglycerides. **(A)** fasting glucose, **(B)** HbA1c, **(C)** triglycerides, and **(D)** TyG index before (week 0) and after 4 and 8 weeks of intervention. Letters in the graph denote *p* < 0.05: *a* compared to baseline (week 0); *b* to control group and *c* to four weeks within the group and were determined by Two-way ANOVA followed by Sìdak *post-hoc* test.

Metabolic improvements were evident as early as 4 weeks in the HIIT group. Plasma glucose, HbA1c, and triglycerides were significantly lower in the HIIT group compared to sedentary subjects and baseline measurements (glucose at 4 weeks: mean difference = −30.4 mg·dL^−1^ vs. control, 95% CI: −53.86 to −6.94; *p* = 0.0027; HbA1c at 4 weeks: mean difference = −3.10%, 95% CI: −5.16 to −1.04; *p* = 0.0002; triglycerides at 4 weeks: mean difference = −39.3 mg·dL^−1^, 95% CI: −74.57 to −4.03; *p* = 0.0173) (Figures 5A,C). At 8 weeks, fasting glucose levels decreased by 32% (−49.8 mg·dL^−1^, 95% CI: −73.26 to −26.34; *p* < 0.0001), HbA1c levels by 35% (−3.30%, 95% CI: −5.36 to −1.24; *p* < 0.0001), and triglycerides by 39% (−70.8 mg·dL^−1^, 95% CI: −106.1 to −35.53; *p* < 0.0001) (Figures 5A,C). According to Guerrero-Romero coworkers (35, 36), all subjects enrolled in our study were insulin resistant since the TyG index was above the 4.5 cutoff level. Eight weeks of HIIT decreased the TyG index by 12%, reaching values close to the cutoff [mean difference = −0.61, 95% CI: −1.06 to −0.16; *p* = 0.0016; from (RED) 6.63 ± 0.7 to 6.02 ± 0.3] (Figure 5D). In contrast, glucose increased in the control group compared to baseline levels (+25.6 mg·dL^−1^, 95% CI: 2.14–49.06; *p* = 0.0216) (Figure 5A). HbA1c, triglycerides, and TyG index remained the same in the control group during the study.

### Metabolic syndrome severity (metS-Z)

**Figure 6:**
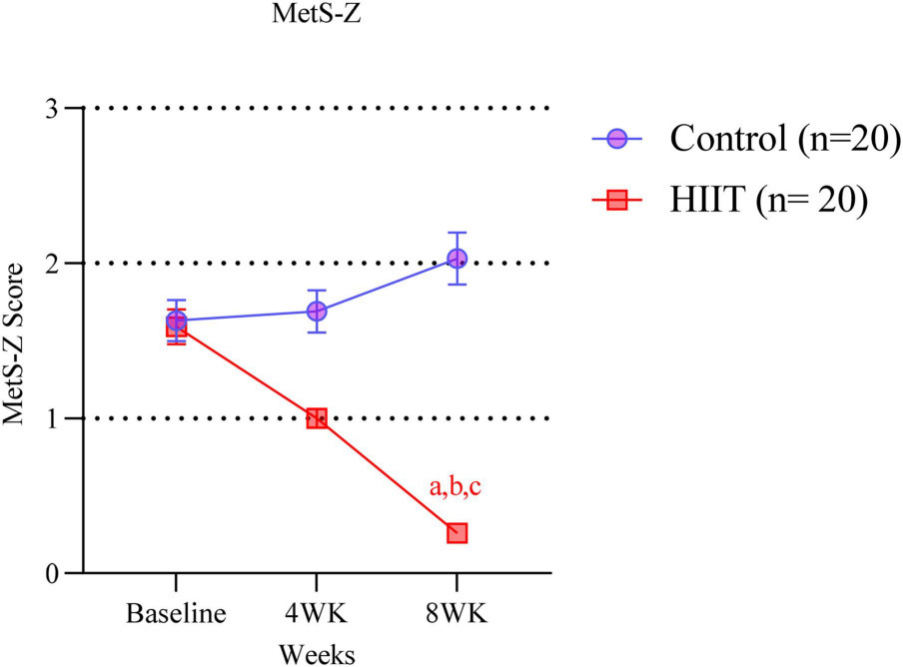
HIIT improves the MetS-Z Score. Letters in the graph denote *p* < 0.05: a compared to baseline (week 0); b to control group and c to four weeks within the group, determined by Two-way ANOVA followed by Sìdak *post-hoc* test.

At baseline, both the control and HIIT groups presented comparable MetS-Z scores (mean ± SD: 1.63 ± 0.59 vs. 1.59 ± 0.50, respectively), indicating elevated cardiometabolic risk in both groups prior to intervention (baseline control vs. HIIT: mean difference = 0.04, 95% CI: −0.45 to 0.53; *p* > 0.9999). Over time, while the MetS-Z did not change in the control group, it progressively decreased in the HIIT after four and eight weeks (Figure 6). The MetS-Z went from 1.59 ± 0.50 to 1.00 ± 0.27 at 4 weeks (mean difference vs. baseline = −0.59, 95% CI: −1.08 to −0.10; *p* = 0.0071) and further to 0.26 ± 0.093 at 8 weeks (mean difference vs. baseline = −1.33, 95% CI: −1.82 to −0.84; *p* < 0.0001) (Figure 6), representing a 84% reduction. At both time points, MetS-Z values were significantly lower in the HIIT group compared to controls (4 weeks: mean difference = −0.69, 95% CI: −1.18 to −0.20; *p* = 0.0008; 8 weeks: mean difference = −1.77, 95% CI: −2.26 to −1.28; *p* < 0.0001).

## Discussion

The present study aimed to determine whether a low-cost, easy-to-implement HIIT protocol based on calisthenics exercise promotes metabolic and cardiovascular health benefits in old individuals with MetS. Over the 8-week intervention, HIIT decreased both systolic and diastolic blood pressures (Figure 3), induced hematologic improvements (Figure 4), decreased plasma glucose and lipid levels, and improved insulin resistance (TyG index; Figure 5). Together, our data reflects in the metabolic syndrome severity marked by MetS-Z score (Figure 6). These positive outcomes were achieved with only three 40-min weekly exercise sessions, without specialized equipment, representing a safe, affordable, and efficient approach to improving health in aged adults. Importantly, the HIIT group reported no adverse effects beyond minor muscle soreness in the first two weeks, and there were no complaints of joint pain or cardiovascular issues. The use of bodyweight exercises ensures feasibility in various settings, such as homes, parks, or community centers. Incorporating such programs into public health initiatives could address the growing prevalence of metabolic syndrome and its associated risks, especially in underserved populations.

To mitigate the adverse impacts of MetS on quality of life, it is essential for exercise programs to improve both insulin resistance and glucose metabolism. HIIT is potentially safe for the general population (36), patients with type 1 (37–39) and type 2 diabetes (40), MetS (41), and obesity (42). HIIT provides acute and chronic benefits for glucose regulation (18, 20, 43), and studies have shown that exercise intensity is a critical factor in HIIT's efficacy for enhancing insulin sensitivity. Indeed, a recent meta-analysis showed that HIIT is superior to MICT in managing glucose levels in people with impaired glucose metabolism and middle-aged adults with T2D (18, 21). Our HIIT protocol significantly enhanced glucose metabolism, evidenced by reductions in fasting glucose and HbA1c. In addition, lowered triglycerides in plasma and consequently decreased the TyG index were documented. As a practical indicator of insulin resistance, TyG is particularly valuable in populations at risk for MetS and diabetes (44, 45). Our results align with findings that HIIT ameliorates insulin sensitivity and promotes better glycemic control in patients suffering from metabolic diseases (25, 46–49).

HIIT substantially increased exercise capacity, with a remarkable 20% improvement in VO_2max_ after 8 weeks in our study. Although our 20% increase in VO_2_max is higher than typically reported in the literature (∼6%) (50), methodological differences such as age distribution, baseline fitness levels, and use of bodyweight calisthenics may explain this discrepancy. Increased VO_2max_ and cardiorespiratory fitness are crucial for disease prevention, and previous studies reported HIIT as one of the most effective exercise modalities to enhance these parameters in various populations, including people with type 2 diabetes (11, 12, 40, 51). For instance, Simonsson et al., demonstrated that 8 weeks of supra-maximal HIIT improved VO_2max_ by 5.9% in old subjects (50). Compared to traditional HIIT using treadmills or cycle ergometers, bodyweight-based HIIT appears to elicit comparable cardiometabolic adaptations (18, 22, 23). The practical advantage is the feasibility in low-resource settings without the need for specialized equipment (52). For example, a 12-week study in adolescents found that a body-weight HIIT program improved cardiorespiratory fitness by ≈4.5 mL/kg/min vs. running HIIT program using treadmills (≈3.3 mL/kg/min**)** (60). Similarly, in adults, 4 weeks of Tabata-style body-weight HIIT (using calisthenic exercises) increased VO_2_max by about 11%**,** which was statistically equivalent to the ∼13% gain achieved with a conventional running-interval HIIT protocol (61). These findings show that body-weight HIIT can match the performance benefits of equipment-centric HIIT**,** while also improving muscle endurance and strength due to the resistance inherent in calisthenics output (53).

The reductions in systolic and diastolic blood pressures within two months are particularly notable given the heightened cardiovascular risk in individuals with MetS. These improvements, along with positive changes in hematologic parameters, suggest that HIIT effectively promotes cardiovascular health in this population. The observed decrease in resting heart rate (RHR) supports prior research findings on HIIT's role in reducing cardiac load, thereby lowering risks of cardiovascular events. This finding is consistent with evidence that HIIT lowers resting heart rate and cardiac workload in hypertensive and cardiometabolic populations (12). Although blood pressure values post-intervention did not fall within the general population's recommended levels (SBP < 120 mmHg and DBP < 80 mmHg), it is plausible to speculate that extending the HIIT program could further reduce these metrics to normal ranges. Meta-analyses of 8-week exercise interventions and studies of extended training programs show significant blood pressure reductions, suggesting that longer HIIT interventions may yield even greater benefits (54–56).

Elevated blood pressure dramatically increases the risk of cardiovascular diseases and death. Hypertension is closely linked to hematological and rheological alterations that affect blood viscosity erythrocyte deformability (ED) and endothelial function (57). The observed hematological adaptations following eight weeks of HIIT, particularly the reduction in hematocrit and mean corpuscular volume (MCV), may contribute to improved cardiovascular function and reductions in arterial pressure (58). Similarly, a decline in MCV has been associated with compensatory mechanisms that enhance blood flow and reduce thrombogenic risk, particularly in hypertensive patients (59). Concomitantly to a reduction in hematocrit, we observed an increase in the amount of hemoglobin. This apparent paradox may reflect plasma volume expansion. Similar patterns of reduced hematocrit accompanied by increased hemoglobin have previously been reported previously (58).

Finally, the severity of the MetS decreased. At baseline, both groups exhibited high MetS-Z scores, but only the HIIT group achieved an 84% reduction (from 1.59 ± 0.50 to 0.26 ± 0.093) after eight weeks. This decrease far exceeds the 10%–25% typically observed in lifestyle or moderate-intensity interventions (62) and surpasses the modest 0.2–0.3 unit changes linked to reduced cardiometabolic risk (37). Compared with aerobic interval training (22) or cycling HIIT (63), which yielded 20%–40% reductions over longer durations, our calisthenics-based HIIT elicited faster, greater improvements, likely driven by concurrent benefits in glucose, triglycerides, and blood pressure. These findings suggest that bodyweight HIIT may offer a more efficient, low-cost strategy for reducing cardiometabolic risk in resource-limited settings.

Mechanistically, this may be due to the combination of improvements we documented in glucose, triglycerides, and blood pressure, which together drive the composite severity index downward. These findings support the notion that HIIT, regardless of modality, can reduce integrated cardiometabolic risk, but our data indicate that bodyweight HIIT may be especially effective in accelerating improvements**,** with implications for its application in resource-limited or community settings.

Our present study has important clinical applications. However, this study has limitations. First, the intervention lasted only 8 weeks, which restricts extrapolation to long-term outcomes. Second, the sample was recruited from a single geographic region in Brazil, limiting generalizability to broader populations. Third, participants were advised but not required to standardize their diet, and no dietary intervention was applied, which may have influenced glucose and lipid outcomes. Finally, physical activity outside the protocol was not strictly monitored, although participants were encouraged to avoid structured additional training.

## Conclusion

This study demonstrates that an accessible, bodyweight HIIT protocol can significantly improve key cardiometabolic health markers in the older adult suffering from metabolic syndrome. The observed reductions in glycemia, insulin resistance, blood pressure, and the improvement on hematological parameters, coupled with increased VO_2max_, highlight the protocol's effectiveness and feasibility.

These findings emphasize the importance of employing it in community health programs, particularly in resource limited settings. However, ensuring the safety and proper implementation of HIIT in older adults is crucial. Due to this, pre-intervention medical screening and continuous monitoring of participants' conditions before, during, and after sessions help maintain appropriate safety levels and reduce risk. Available evidence indicates that HIIT can be safe and feasible in older populations when adequately supervised and individually adapted, although data on adverse events remain limited. Therefore, we recommend routine pre-participation health assessment, individualized intensity prescription, and ongoing cardiovascular monitoring throughout HIIT interventions in older adults to optimize safety and effectiveness.

## Data Availability

The raw data supporting the conclusions of this article are available from the authors upon request.
